# A Facile Synthesis of New 2-Amino-4*H*-pyran-3-carbonitriles by a One-Pot Reaction of **α**, **α**′-Bis(arylidene) Cycloalkanones and Malononitrile in the Presence of K_2_CO_3_

**DOI:** 10.1100/2012/208796

**Published:** 2012-04-01

**Authors:** Zahed Karimi-Jaberi, Baharak Pooladian

**Affiliations:** Department of Chemistry, Firoozabad Branch, Islamic Azad University, P.O. Box 74715-117 Firoozabad, Fars, Iran

## Abstract

A rapid and environmentally friendly method is developed for the synthesis of a series of new substituted 2-amino-4*H*-pyran-3-carbonitriles through a one-pot condensation of malononitrile and *α*, *α*′-bis(arylidene) cycloalkanones in ethanol by using K_2_CO_3_ as a catalyst. Short experimental reaction times, excellent yields, no need to use cumbersome apparatus for purification of the products, and inexpensiveness and commercially availability of the catalyst are the advantages of this method.

## 1. Introduction

In the past few decades, the synthesis of new heterocyclic compounds has been a subject of great interest due to the wide applicability of them. Heterocyclic compounds occur very widely in nature and are essential to life. The importance of multicomponent reactions in organic synthesis has been recognized, and considerable efforts have been focused on the design and development of one-pot procedures for the generation of libraries of heterocyclic compounds [1, 2]. 4*H*-Pyrans and their derivatives are of considerable interest due to their pharmacological activities [3], such as spasmolytic, diuretic, anticoagulant, anticancer, and antianaphylactic activity [4–6]. Moreover, 4*H*-pyrans are useful intermediates for synthesis of various compounds, such as pyranopyridine derivatives [7], polyazanaphthalenes [8], pyranopyrimidines [9], and pyridin-2-ones [10]. 

**Scheme 1:**
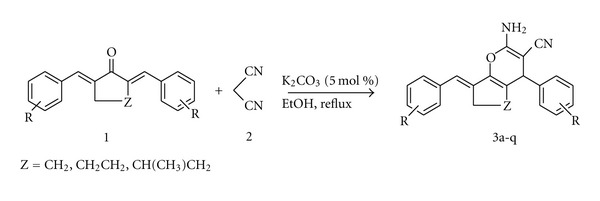
Synthesis of 2-amino-4*H*-pyran-3-carbonitriles.

Furthermore, 4*H-*pyrans represent building blocks of a series of natural products [11, 12]. A number of 2-amino-4*H*-pyrans are used as photoactive materials [13], pigments [14], and potential biodegradable agrochemicals [15], and consequently, numerous methods have been reported for the synthesis of these compounds. Thus, the synthesis of 4*H*-pyran is of much importance to organic chemists. Several methods have been reported for the synthesis of pyran derivatives via a three-component condensation of *β*-dicarbonyl compounds with aldehydes and malononitrile [16]. From the literature, we observed that very few catalysts have been used for the synthesis of 2-amino-4*H*-pyran-3-carbonitriles base on the reactions of *α*, *α*′-bis(arylidene) cycloalkanones with malononitrile, for example, NaOH/piperidine [17], KF-Al_2_O_3_ [18], and hexadecyltrimethyl ammonium bromide (HTMAB) [19]. However, these methods show varying degrees of success as well as limitations such as prolonged reaction times, low yields, and use of toxic solvents. Thus, the development of an alternate milder and clean procedure is highly demanding for the synthesis of 2-amino-4*H*-pyran-3-carbonitriles, which surpasses those limitations. Herein, we planned to synthesis of these compounds using sequential reactions of *α*, *α*′-bis(arylidene) cycloalkanones and malononitrile in the presence of K_2_CO_3_ as a catalyst in ethanol under reflux conditions (Scheme 1). 

Nowadays, organic reactions in ethanol without the use of harmful organic solvents have attracted much attention, because ethanol is a cheap, safe, and environmentally benign solvent [7]. In recent years, K_2_CO_3_ has been considered as an efficient, inexpensive, and readily available catalyst for several organic transformations [20, 21].

## 2. Results and Discussion 

In continuation of our studies on the development of inexpensive and environmentally benign methodologies for organic reactions [22–24], herein we report a highly versatile and efficient synthesis of 2-amino-4*H*-pyran-3-carbonitriles **3a–q** (Scheme 1) from *α*, *α*′-bis(Arylidene) cycloalkanone **1**, malononitrile **2** and catalytic amounts of K_2_CO_3_. In a typical reaction, a mixture of **1** and **2 **(1 : 1) equivalents, respectively, and K_2_CO_3_ (cat.) was refluxed in ethanol for 5–60 min. The results are summarized in (Table 1). 

**Scheme 2:**
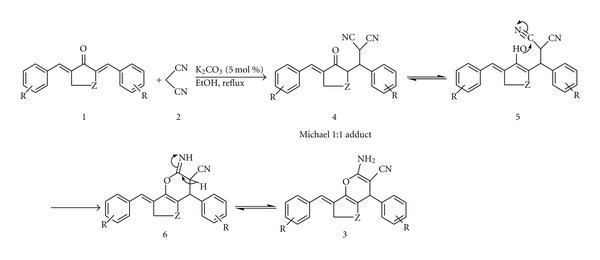
Proposed mechanism.

The formation of the compounds **3** was assumed to proceed via formation of a Michael adduct intermediate followed by cyclization according to Scheme 2. A *α*, *α*′-bis(arylidene) cycloalkanones **1** was firstly condensed with malononitrile **2** to afford the intermediate **4**, this step can be regarded as a Michael addition. Then, the intermediate **5** cyclized by nucleophilic attack of the OH group on the cyano (CN) moiety and gave the intermediate **6**. Finally, the expected products **3** were afforded (Scheme 2) [17–19]. 

To test the catalysts, the reaction of *α*, *α*′-bis(arylidene) cyclohexanone and malononitrile in ethanol was selected as a model reaction. The scope and the generality of the present method were then further demonstrated by the reaction of various *α*, *α*′-bis(arylidene) cycloalkanones with malononitrile and K_2_CO_3_. In all cases, good yields with good selectivity were obtained. The catalyst plays a crucial role in the success of the reaction in terms of the rate and the yields. The present methodology afforded high yields of the products within short times (5–60 min). The results (Table 1, entries 1–17) indicated that substrates **1 **bearing both electron-donating groups (such as alkoxy and methyl) and electron-withdrawing groups (such as halide) can be involved in this one-pot synthesis to afford desired products **3 **with high yields. Thus, it should be concluded that the electronic nature of the substituents has no significant effect on this reaction. 

In order to show the merits of K_2_CO_3_ over other catalysts reported in the literature, results for the synthesis of 2-amino-4*H*-pyran-3-carbonitriles obtained using K_2_CO_3_ as the catalyst were compared with those obtained using other catalysts.  Table 2 clearly shows that K_2_CO_3_ appears to promote the reaction more effectively than a number of other catalysts, particularly in terms of the time and yield required to complete the reaction. 

## 3. Conclusion

In conclusion, the present method is a simple and environmentally friendly procedure for the synthesis of a series of new 2-amino-4*H*-pyran-3-carbonitriles using catalytic amount of K_2_CO_3_. The simple experimental procedure, short reaction times, excellent yields of products, mild reaction condition, easy purification, economic availability of the catalyst, and green standard are the advantages of this method.

## 4. Method

*α*, *α*′-Bis(arylidene)cycloalkanones have been synthesized through cross-aldol condensation of cycloalkanones and aldehydes using our reported method [25]. 

### 4.1. General Procedure for Synthesis of 2-Amino-4*H*-pyran-3-carbonitrile Derivatives 3a–q

A mixture of appropriate *α*, *α*′-bis(arylidene)cycloalkanone **1 **(1 mmol), malononitrile **2 **(1 mmol) and 5% mol K_2_CO_3_ (0.05 mmol, 0.006 g) in ethanol 96% (10 mL) was refluxed for the appropriate time indicated in Table 1 (5–60 min). The progress of the reaction was monitored by TLC. After completion of the reaction, the reaction mixture was cooled to room temperature, and the resulting cream precipitate was filtered and washed with *n*-hexane (10 mL) to furnish the corresponding 2-amino-4*H*-pyran-3-carbonitriles. 

The structure of the products was deduced from their IR, ^1^H NMR, ^13^C NMR, and elemental analysis. The spectral (IR, ^1^H NMR, ^13^C NMR) and analytical data of unknown compounds are given below.

#### 4.1.1. 8-(4-fluorobenzylidene)-2-amino-4-(4-fluorophenyl)-5,6,7,8-tetrahydro-4*H*-chromene-3-carbonitrile (Entry 7, **3 g**)

Cream powder, IR(KBr): 3465, 3342, 2945, 2196, 1671, 1643, 1599, 1503, 1414, 1221, 1134, 1029, 834, 803 cm^−1^; ^1^HNMR (250 MHz, CDCl_3_): *δ* = 1.59–1.63 (m, 2H, CH_2_), 1.85–2.04 (m, 2H, CH_2_), 2.50–2.71 (m, 2H, CH_2_), 3.95 (s, 1H, CH), 4.55 (s, 2H, NH_2_), 6.82 (s, 1H, =CH), 6.99–7.07 (m, 4H, ArH), 7.18–7.28 (m, 4H, ArH); ^13^CNMR (62.9 MHz, CDCl_3_): *δ* = 22.17, 26.96, 27.37, 42.88, 60,44, 115.03, 115.36, 115.50, 115.84, 119.77, 121.37, 129.35, 129.48, 130.75, 130.88, 138.61, 141.40, 158.08, 151.80. Anal. Calcd For C_23_H_18_F_2_N_2_O: C, 73.39; H, 4.82; N, 7.44; Found: C, 73.25; H, 4.79; N, 7.40.

#### 4.1.2. 8-(4-bromobenzylidene)-2-amino-4-(4-bromophenyl)-5,6,7,8-tetrahydro-4*H*-chromene-3-carbonitrile (Entry 8, **3 h**)

Cream powder, IR(KBr): 3443, 3318, 3212, 2194, 1665, 1635, 1416, 1126, 1007, 821 cm^−1^; ^1^HNMR (250 MHz, CDCl_3_): *δ* = 1.61–1.79 (m, 2H, CH_2_), 1.84–2.16 (m, 2H, CH_2_), 2.49–2.94 (m, 2H, CH_2_), 3.93 (s, 1H, CH), 4.56 (s, 2H, NH_2_), 6.79 (s, 1H, =CH), 7.16–7.23 (m, 4H, ArH), 7.45–7.48 (m, 4H, ArH); ^13^CNMR (62.9 MHz, CDCl_3_): *δ* = 22.11, 27.0, 27.37, 43.14, 60.03, 115.14, 120.82, 121.36, 121.80, 121.85, 129.62, 129.86, 130.80, 131.36, 131.95, 135.78, 141.51, 141.80, 158.86. Anal. Calcd For C_23_H_18_Br_2_N_2_O: C, 55.45; H, 3.64; N, 5.62; Found: C, 55.34; H, 3.60; N, 5.59.

#### 4.1.3. 8-(4-methoxybenzylidene)-2-amino-4-(4-methoxyphenyl)-5,6,7,8-tetrahydro-4*H*-chromene-3-carbonitrile (Entry 10, **3 j**)

Cream powder, IR(KBr): 3446, 3335, 2925, 2836, 2188, 1667, 1630, 1603, 1508, 1404, 1249, 1127, 1029, 831 cm^−1^; ^1^HNMR (250 MHz, CDCl_3_): *δ* = 1.34–1.62 (m, 2H, CH_2_), 1.94–1.96 (m, 2H, CH_2_), 2.53–2.91 (m, 2H, CH_2_), 3.79 (s, 3H, OCH_3_), 3.82 (s, 3H, OCH_3_), 3.91(s, 1H, CH), 4.51 (s, 2H, NH_2_), 6.81 (s, 1H, =CH), 6.85–6.91 (m, 4H, ArH), 7.14–7.26 (m, 4H, ArH); ^13^CNMR (62.9 MHz, CDCl_3_): *δ* = 22.29, 27.12, 27.35, 27.40, 42.76, 55.27, 60.81, 113.66, 114.10, 114.74, 122.02, 127.95, 128.92, 129.60, 129.70, 130.54, 135.10, 135.15, 141.42, 158.41, 158.81. Anal. Calcd For C_25_H_24_N_2_O_3_: C, 74.98; H, 6.04; N, 7.0; Found: C, 75.01; H, 6.07; N, 7.04.

#### 4.1.4. 8-(2-chloro-6-fluorobenzylidene)-2-amino-4-(2-chloro-6-fluorophenyl)-5,6,7,8-tetrahydro-4*H*-chromene-3-carbonitrile (Entry 12, **3 l**)

Cream powder, IR(KBr): 3456, 3339, 2944, 2913, 2188, 1672, 1637, 1597, 1443, 1413, 1240, 1130, 897, 780, 756 cm^−1^; ^1^HNMR (250 MHz, CDCl_3_): *δ* = 1.59–1.65 (m, 2H, CH_2_), 1.93–2.11 (m, 2H, CH_2_), 2.25–2.86 (m, 2H, CH_2_), 4.60 (s, 1H, CH), 4.89 (s, 2H, NH_2_), 6.56 (s, 1H, =CH), 6.84–7.03 (m, 2H, ArH), 7.14–7.25 (m, 4H, ArH); ^13^CNMR (62.9 MHz, CDCl_3_): *δ* = 21.80, 27.15, 27.56, 43.10, 58.01, 113.26, 113.89, 114.27, 119.60, 124.11, 124.39, 124.76, 125.10, 128.76, 128.91, 129.17, 129.33, 132.56, 133.53, 134.72, 135.05, 158.41, 160.13. Anal. Calcd For C_23_H_16_C_12_F_2_N_2_O: C, 62.04; H, 3.62; N, 6.29; Found: C, 62.08; H, 3.64; N, 2.23.

#### 4.1.5. 2-amino-8-benzylidene-6-methyl-4-phenyl-5,6,7,8-tetrahydro-4*H*-chromene-3-carbonitrile (Entry 13, **3 m**)

Cream powder, IR(KBr): 3433, 3329, 2925, 2910, 2190, 1670, 1632, 1594, 1485, 1409, 1009 cm^−1^; ^1^HNMR (250 MHz, CDCl_3_): *δ* = 0.90 (d, 3H, *J *= 6.2 Hz, CH_3_), 1.60–2.27 (m, 4H, 2CH_2_), 2.81–2.87 (m, 1H, CH), 3.94 (s, 1H, CH), 4.49 (s, 2H, NH_2_), 6.88 (s, 1H, =CH), 7.22–7.37 (m, 10H, ArH); ^13^CNMR (62.9 MHz, CDCl_3_): *δ* = 21.0, 28.55, 34.76, 35.09, 43.09, 61.01, 114.62, 119.20, 120.0, 122.86, 126.82, 127.36, 127.90, 128.22, 128.82, 129.27, 137.01, 142,13, 143.01, 158.93. Anal. Calcd For C_24_H_22_N_2_O: C, 81.33; H, 6.26; N, 7.90; Found: C, 81.40; H, 6.23; N, 7.95.

#### 4.1.6. 8-(2-chlorobenzylidene)-2-amino-4-(2-chlorophenyl)-6-methyl-5,6,7,8-tetrahydro-4*H*-chromene-3-carbonitrile (Entry 14, **3 n**)

Cream powder, IR(KBr): 3472, 3330, 2945, 2924, 2192, 1674, 1635, 1594, 1411, 1130, 1036, 738 cm^−1^; ^1^HNMR (250 MHz, CDCl_3_): *δ* = 0.85 (d, 3H, *J *= 6.2 Hz, CH_3_), 1.53–1.87 (m, 2H, CH_2_), 1.96–2.17 (m, 2H, CH_2_), 2.59–2.65 (m, 1H, CH), 4.62 (s, 1H, CH), 4.69 (s, 2H, NH_2_), 6.92 (s, 1H, =CH), 7.25–7.43 (m, 8H, ArH); ^13^CNMR (62.9 MHz, CDCl_3_): *δ* = 20.90, 28.81, 35.10, 35.55, 39.69, 59.24, 119.63, 120.19, 126.27, 127.61, 128.30, 128.59, 129.49, 129.77, 130.46, 130.68, 133.48, 134.08, 135.24, 135.55, 139.84, 140.89, 141.31, 159.36. Anal. Calcd For C_24_H_20_Cl_2_N_2_O: C, 68.09; H, 4.76; N, 6.62; Found: C, 68.0; H, 4.78; N, 6.58.

#### 4.1.7. 8-(4-chlorobenzylidene)-2-amino-4-(4-chlorophenyl)-6-methyl-5,6,7,8-tetrahydro-4*H*-chromene-3-carbonitrile (Entry 15, **3 o**)

Cream powder, IR(KBr): 3423, 3329, 2925, 2190, 1672, 1636, 1590, 1483, 1409, 1126, 1010, 823 cm^−1^; ^1^HNMR (250 MHz, CDCl_3_): *δ* = 0.88 (d, 3H, *J *= 6.0 Hz, CH_3_), 1.55–2.45 (m, 4H, 2CH_2_), 2.75–2.80 (m, 1H, CH), 3.93 (s, 1H, CH), 4.62 (s, 2H, NH_2_), 6.82 (s, 1H, =CH), 7.14–7.34 (m, 8H, ArH); ^13^CNMR (62.9 MHz, CDCl_3_): *δ* = 20.97, 28.86, 34.64, 35.07, 43.39, 60.05, 114.36, 114.48, 119.76, 121.85, 128.43, 128.98, 129.29, 130.52, 132.64, 135.34, 141.16, 141.56, 158.90, 158.99. Anal. Calcd For C_24_H_20_Cl_2_N_2_O: C, 68.09; H, 4.76; N, 6.62; Found: C, 68.07; H, 4.78; N, 6.64.

#### 4.1.8. 8-(4-methyelbenzylidene)-2-amino-4-(4-metheylyphenyl)-6-methyl-5,6,7,8-tetrahydro-4*H*-chromene-3-carbonitrile (Entry 16, **3 p**)

Cream powder, IR(KBr): 3441, 3350, 2960, 2944, 2194, 1675, 1639, 1598, 1411, 1131, 809 cm^−1^; ^1^HNMR (250 MHz, CDCl_3_): *δ* = 0.91 (d, 3H, *J *= 6.7 Hz, CH_3_), 1.64–2.08 (m, 4H, 2CH_2_), 2.34 (s, 3H, CH_3_), 2.36 (s, 3H, CH_3_), 2.81–2.87 (m, 1H, CH), 3.91 (s, 1H, CH), 4.50 (s, 2H, NH_2_), 6.83 (s, 1H, =CH), 7.12–7.32 (m, 8H, ArH); ^13^CNMR (62.9 MHz, CDCl_3_): *δ* = 21.06, 21.14, 21.24, 29.01, 35.24, 36.11, 43.51, 60.68, 114.28, 114.37, 122.49, 127.83, 128.95, 129.22, 129.45, 134.13, 136.63, 136.97, 139.73, 140.13, 141.19, 158.79. Anal. Calcd For C_26_H_26_N_2_O: C, 81.64; H, 6.85; N, 7.32; Found: C, 81.57; H, 6.86; N, 7.34.

#### 4.1.9. 8-(4-methoxybenzylidene)-2-amino-4-(4-methoxyphenyl)-6-methyl-5,6,7,8-tetrahydro-4*H*-chromene-3-carbonitrile (Entry17, **3 q**)

Cream powder, IR(KBr): 3461, 3323, 2924, 2846, 2194, 1671, 1635, 1593, 1417, 1124, 845 cm^−1^; ^1^HNMR (250 MHz, CDCl_3_): *δ* = 0.88 (d, 3H, *J *= 5.7 Hz, CH_3_), 1.60–2.17 (m, 4H, 2CH_2_), 2.81–2.87 (m, 1H, CH), 3.80 (s, 3H, OCH_3_), 3.83 (s, 3H, OCH_3_), 3.90 (s, 1H, CH), 4.51 (s, 2H, NH_2_), 6.81 (s, 1H, =CH), 6.85–6.92 (m, 4H, ArH), 7.13–7.26 (m, 4H, ArH); ^13^CNMR (62.9 MHz, CDCl_3_): *δ* = 21.08, 28.50, 28.97, 35.25, 36.06, 43.09, 55.28, 60.83, 113.68, 114.09, 120, 122.10, 127.31, 128.40, 128.95, 129.56, 130.56, 135.10, 141.10, 158.42, 159.24, 159.83. Anal. Calcd For C_26_H_26_N_2_O_3_: C, 75.34; H, 6.32; N, 6.76; Found: C, 75.37; H, 6.28; N, 6.73.

## Figures and Tables

**Table 1:** Synthesis of 2-amino-4*H*-pyran-3-carbonitriles 3a–q^a^.

Entry	Z	R	Product	Time (min)	Yield (%)^b^	mp (**°**C)	Ref
1	CH_2_	H	** 3a**	45	87	227-228	[19]
2	CH_2_	2-Cl	**3b**	5	90	213-214	[19]
3	CH_2_	2,4-Cl_2_	**3c**	15	93	238-239	[18]
4	CH_2_-CH_2_	H	**3d**	10	95	228–230	[17]
5	CH_2_-CH_2_	2-Cl	**3e**	10	85	237-238	[19]
6	CH_2_-CH_2_	4-Cl	**3f**	15	85	215-216	[19]
7	CH_2_-CH_2_	4-F	**3g**	10	90	222–224	—
8	CH_2_-CH_2_	4-Br	**3h**	15	88	214–217	—
9	CH_2_-CH_2_	4-Me	**3i**	60	90	161-162	[19]
10	CH_2_-CH_2_	4-OMe	**3j**	10	80	220–222	—
11	CH_2_-CH_2_	2,4-Cl_2_	**3k**	15	87	195-196	[18]
12	CH_2_-CH_2_	2-Cl, 6-F	**3l**	10	85	233–236	—
13	CH(CH_3_)CH_2_	H	**3m**	20	90	199–202	—
14	CH(CH_3_)CH_2_	2-Cl	**3n**	25	87	198–201	—
15	CH(CH_3_)CH_2_	4-Cl	**3o**	15	85	208-209	—
16	CH(CH_3_)CH_2_	4-Me	**3p**	60	75	214–218	—
17	CH(CH_3_)CH_2_	4-OMe	**3q**	20	80	199–202	—

^a^Reaction conditions:  *α*, *α*′-bis(arylidene) cycloalkanones **1** (1 mmol), malononitrile **2** (1 mmol), K_2_CO_3_ (0.05 mmol, 5 mol%), EtOH (10 mL), reflux. ^b^Isolated yields.

**Table 2:** Comparison of results using K_2_CO_3_ with other catalyst for synthesis of 2-amino-4*H*-pyran-3-carbonitriles.

Entry	Catalyst	Solvent	T	Time	Yield (%)	Ref
1	NaOH/Piperidine	EtOH	MW	5–9 h	70-71	[17]
2	KF-Al_2_O_3_	DMF	R·T	10–14 h	68–90	[18]
3	HTMAB	H_2_O	110°C	8 h	76–93	[19]
4	K_2_CO_3_	EtOH	Reflux	5–60 min	75–95	This work
